# Effect of artificial aging on fracture toughness and hardness of 3D‐printed and milled 3Y‐TZP zirconia

**DOI:** 10.1111/jopr.13943

**Published:** 2024-09-03

**Authors:** Abdulaziz Alhotan, Burak Yilmaz, Anna Weber, Rua Babaier, Christoph Bourauel, Ahmed Mahmoud Fouda

**Affiliations:** ^1^ Department of Dental Health College of Applied Medical Sciences King Saud University Riyadh Saudi Arabia; ^2^ Department of Reconstructive Dentistry and Gerodontology School of Dental Medicine University of Bern Bern Switzerland; ^3^ Department of Oral Technology Medical Faculty University Hospital Bonn Bonn North Rhine‐Westphalia Germany; ^4^ Department of Prosthetic Dental Sciences College of Dentistry King Saud University Riyadh Saudi Arabia; ^5^ Department of Fixed Prosthodontics Suez Canal University Ismailia Egypt

**Keywords:** 3D‐printed 3Y‐TZP, aging, fracture toughness, hardness, lithography‐based ceramic manufacturing, milled 3Y‐TZP

## Abstract

**Purpose:**

This study aimed to evaluate the impact of artificial aging on the fracture toughness and hardness of three‐dimensional (3D)‐printed and computer‐aided design and computer‐aided manufacturing (CAD‐CAM) milled 3 mol% yttria‐stabilized tetragonal zirconia polycrystals (3Y‐TZP).

**Materials and Methods:**

Forty bar‐shaped specimens (45 × 4 × 3 mm) were prepared using two manufacturing technologies: 3D printing (LithaCon 3Y 210, Lithoz GmbH, Vienna, Austria; *n* = 20) and milling (Initial Zirconia ST, GC, Japan; *n* = 20) of 3Y‐TZP. The chevron‐notch beam method was used to assess the fracture toughness according to ISO 24370. Specimens from each 3Y‐TZP group were divided into two subgroups (*n* = 10) based on the artificial aging process (autoclaving): nonaged and aged. Nonaged specimens were stored at room temperature, while aged specimens underwent autoclave aging at 134°C under 2 bar‐pressure for 5 h. Subsequently, the specimens were immersed in absolute 99% ethanol using an ultrasonic cleaner for 5 min. Each specimen was preloaded by subjecting it to a 4‐point loading test, with a force of up to 200 N applied for three cycles. Further 4‐point loading was conducted at a rate of 0.5 mm/min under controlled temperature and humidity conditions until fracture occurred. The maximum force (*F*_max_) was recorded and the chevron notch was examined at 30 × magnification under an optical microscope for measurements before the fracture toughness (*K*_Ic_) was calculated. Microhardness testing was also performed to measure the Vickers hardness number (VHN). A scanning electron microscope (SEM) coupled with an energy dispersive X‐ray unit (EDX) was used to examine surface topography and chemical composition. X‐ray diffraction (XRD) was conducted to identify crystalline structure. Data were statistically analyzed using two‐way ANOVA and Student's t‐test with a significance level of 0.05.

**Results:**

The nonaged 3D‐printed 3Y‐TZP group exhibited a significantly higher fracture toughness value (6.07 MPa m^1/2^) than the milled 3Y‐TZP groups (*p* < 0.001). After autoclave aging, the 3D‐printed 3Y‐TZP group maintained significantly higher fracture toughness (*p* < 0.001) compared to the milled 3Y‐TZP group. However, no significant differences in hardness values (*p* = 0.096) were observed between the aged and nonaged groups within each manufacturing process (3D‐printed and milled) independently.

**Conclusion:**

The findings revealed that the new 3D‐printed 3Y‐TZP produced by the lithography‐based ceramic manufacturing (LCM) technology exhibited superior fracture toughness after autoclave aging compared to the milled 3Y‐TZP. While no significant differences in hardness were observed between the aged groups, the 3D‐printed material demonstrated greater resistance to fracture, indicating enhanced mechanical stability.

Yttrium‐stabilized tetragonal zirconia (Y‐TZP) is extensively used in various dental applications due to its superior mechanical characteristics.^1^ Its high mechanical properties are linked to the transformation toughening mechanism, which involves the conversion of the tetragonal phase to the monoclinic phase when stress occurs near a propagating crack.^2^ Typically, 3Y‐TZP exhibits flexural strength of 900–1400 MPa and fracture toughness values ranging from 5 to 10 MPa m^1/2^, which enables its application in the fabrication of multi‐unit fixed partial dentures (FPDs) within high load‐bearing regions.^3^, ^4^, ^5^ The integration of zirconia in prosthetic and restorative dentistry has been closely connected with advancements in design and manufacturing technologies, specifically computer‐aided design and computer‐aided manufacturing (CAD‐CAM).^1^

Over the past few years, additive manufacturing (AM), another term that describes 3D printing, has been receiving increasing interest over subtractive manufacturing (SM).^6^ This shift is driven by the manifold advantages that additive manufacturing offers, encompassing the possibility to reproduce complex surface geometries, cost‐efficiency, speed processing, reduction in material waste, and exclusion of milling‐associated risks such as crack initiation and wasting of milling tools.^7^ The rapid evolution of innovative 3D printing technologies has ushered in a wave of new 3D‐printed materials, predominantly resin‐based, into the dental market. Within the realm of dentistry, these resin‐based materials find diverse applications, ranging from the production of diagnostic and educational models, surgical guides, customized replacement prostheses, and denture bases, among various other use cases.^8^, ^9^, ^10^, ^11^ Nevertheless, it is crucial to acknowledge that the mechanical properties of these resin‐based materials continue to impose limitations on their utility within the restorative dental field, primarily confining them to provisional applications.

Ongoing trials are progressively integrating high‐strength ceramic materials into the expanding array of materials available for 3D printing. Ceramic materials exhibit distinct advantages over resin‐based materials, encompassing enhanced aesthetics, superior mechanical properties, and biocompatibility. The advent of lithography‐based ceramic manufacturing (LCM) technology, pioneered by Lithoz (Lithoz, Vienna, Austria) has paved the way for the 3D printing of ceramic‐based materials, including zirconia.^12^ LCM relies on direct light processing (DLP), wherein a ceramic slurry containing a photosensitive polymer network is cured in sequential layers by irradiating short‐wavelength light beams ranging from 380 to 405 nm.^13^ Recent investigations have uncovered promising findings regarding 3D‐printed zirconia crowns, particularly regarding their marginal fit^14^ and fracture strength^15^, ^16^ compared to traditionally milled counterparts. LCM was selected for this research due to its ability to produce high‐density ceramic parts with superior surface quality, precision, and mechanical properties, which are critical for dental applications.^17^ When compared to other ceramic 3D printing methods such as selective laser sintering (SLS) and fused deposition modeling (FDM), FDM exhibits significant limitations that hinder mass production due to its lower accuracy, speed, and production capacity, as well as higher production costs. Additionally, the parts produced by SLS are limited by the size of the 3D printers currently available on the market, and the process is less efficient for very large‐scale production due to limited printer volume capacity.^18^ Thus, LCM is the preferred method for producing high‐performance ceramic dental parts.

However, the literature is notably deficient in comprehensive studies pertaining to the toughness properties of 3D‐printed zirconia and the influence of aging on its performance. Understanding these properties is essential because they directly impact the longevity and reliability of dental restorations. Improvements in fracture toughness and hardness can lead to enhanced durability and resistance to wear, which are critical for high‐load‐bearing dental applications. This knowledge can influence clinical practice by guiding the choice of materials and methods in prosthodontics, ultimately improving patient outcomes by providing more reliable and long‐lasting dental restorations.

This study aimed to evaluate the fracture toughness and hardness of 3D‐printed and milled 3Y‐TZP after artificial aging. The null hypothesis was that neither fracture toughness nor hardness would be affected by aging.

## MATERIALS AND METHODS

### Specimen preparation

The calculation for the sample size was performed using the G*Power software program (v3.1.3; Heinrich Heine University Düsseldorf). This calculation was based on a significance level of 0.05, a power probability of 80%, and a medium effect size (Cohen's) of 0.6. The output revealed that nine specimens were required for each test (fracture toughness and hardness) under subgroup (either nonaged or aged); however, for the purposes of this study, ten specimens per group were chosen.

Forty bar specimens (45 × 4 × 3 mm) were designed using online software (Tinkercad, Autodesk, California, USA) following the International Organization Standardization (ISO) 24370.^19^ They were subsequently produced from 3Y‐TZP using two manufacturing technologies: 3D printing and CAD‐CAM milling methods.

For the 3D‐printed group, a CeraFab 8500 printer (Lithoz GmbH, Vienna, Austria) was used to print 20 specimens from LithaCon 3 210 (Lithoz GmbH, Vienna, Austria). Printing parameters were configured as follows: layer thickness of 25 µm, a shrinkage ratio of 1.27 in the *X*/*Y* plane, and a shrinkage ratio of 1.30 in the *Z* direction. After printing, the special cleaning fluid LithaSol 30 (Lithoz, Vienna, Austria) was used to clean the beams of the slurry. Subsequently, the printed specimens underwent a multi‐step heat treatment process within three consecutive furnaces, including preconditioning at 120°C for 134 h, followed by debinding at temperatures of up to 1000°C for 103 h and sintering at temperatures of up to 1450°C for 17 h. For the milled group fabrication, 20 bar‐shaped specimens were milled from Initial Zirconia ST blanks (GC Inc., Tokyo, Japan) and sintered according to the manufacturer's recommendations.

**FIGURE 1:**
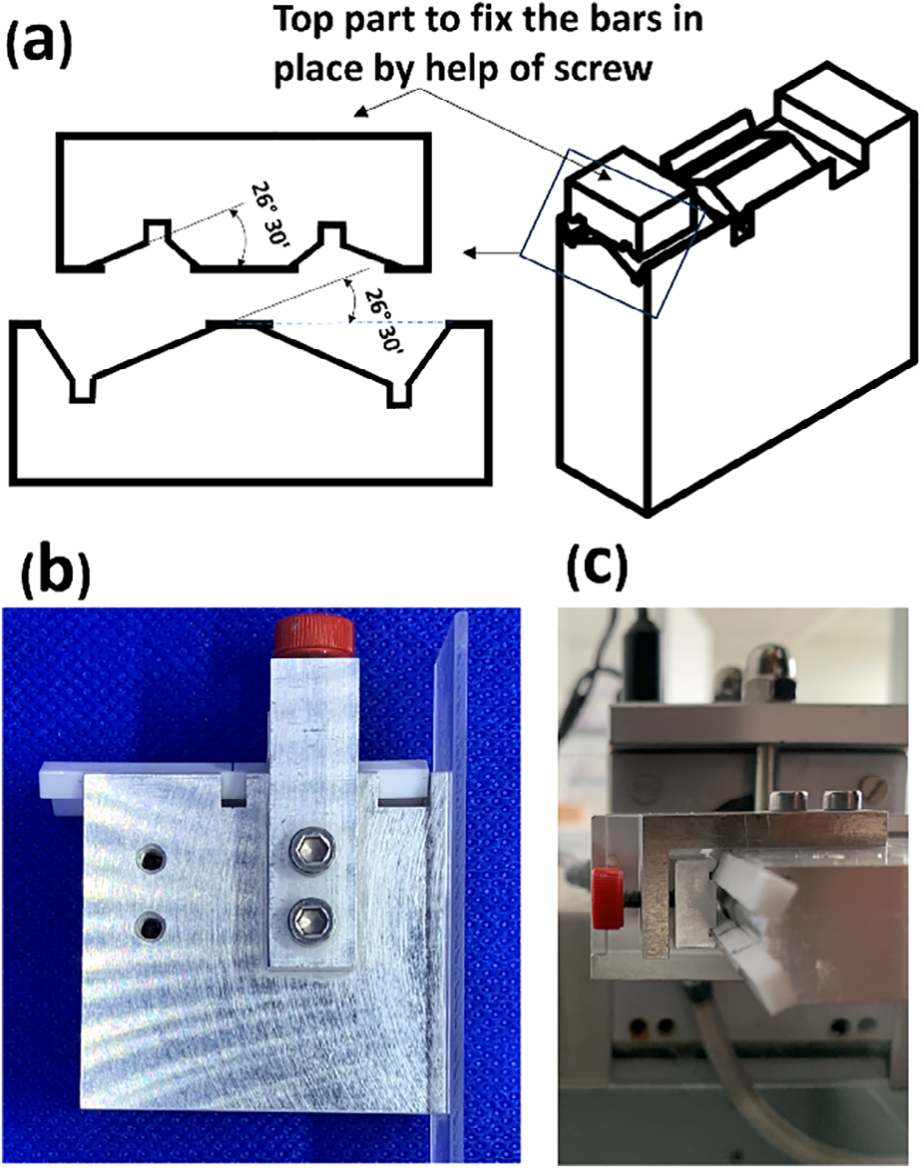
Specially designed specimen holder. (a) A schematic diagram showing the angled planes for bar placement, (b) bars placed in the holder and fixed by the help of the red‐headed screw, (c) the holder is fixed to the cutting machine.

The specimens underwent a rigorous preparation process for fracture toughness assessment within the framework of this scientific study. Polishing was accomplished by employing a micromotor with a polishing tip, operating at a velocity of 250,000 revolutions per minute. During the notch preparation phase, a specially designed holder was utilized to secure the specimens in place (Figure 1). Notches were introduced using a micrometer cutting saw featuring a 1 mm thick saw blade, with two bars being simultaneously affixed to the holder at a 26° angle relative to the saw blade. Following the initial cut, the positions of the two bars were interchanged, and they were rotated 180° around their long axes before executing the second cut. Subsequently, an optical microscope was employed to measure the notch dimensions. Specimens that did not meet the notch dimensions specified by ISO were excluded.

**FIGURE 2:**
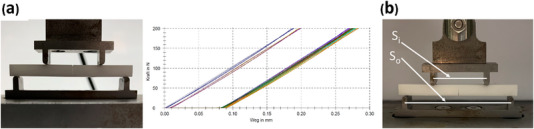
(a) Preloading with three cycles up to 200 N to release residual stresses. The left image shows the notch facing upwards during preloading. The right image represents the Zwick report. (b) 4‐point loading test with the notch facing downward. S_i_ is the flexure fixture inner span in millimeters, S_o_: is the flexure fixture outer span in millimeters.

Each 3Y‐TZP manufacturing technology (*n* = 20) was further divided into two subgroups (*n* = 10) according to the artificial aging process (autoclaving): nonaged and aged subgroups. The nonaged specimens were stored at approximately 23°C. In contrast, the aged specimens underwent autoclave aging at 134°C under 2 bar pressure for 5 h.^20^ Both aged and non‐aged specimens were then immersed in 99% absolute ethanol using an ultrasonic cleaner for 5 min. The specimens were subsequently dried and investigated using an optical microscope to document the notch measurements. Each specimen was preloaded by subjecting it to a four‐point loading test, with a force of up to 200 N applied for three cycles, within a universal testing machine.^19^ This was conducted with the notches facing upward to release any residual stresses (Figure 2a). The specimens, along with silicon oil, were subsequently transferred to a drying oven and exposed to a temperature of 110°C for 1 h. Following this, drops of hot silicon oil were administered to the notches, and the specimens were left to cool for 1 h. The final testing was conducted within 24 h after the specimens were removed from the drying oven.

### Fracture toughness measurement

All specimens were subjected to four‐point loading with the notch oriented downward (Figure 2b). A 5K loading cell was employed, and the specimen was loaded at a rate of 0.05 mm/min under controlled temperature and humidity conditions until fracture occurred. The maximum force (*F*_max_) was recorded.

**FIGURE 3:**
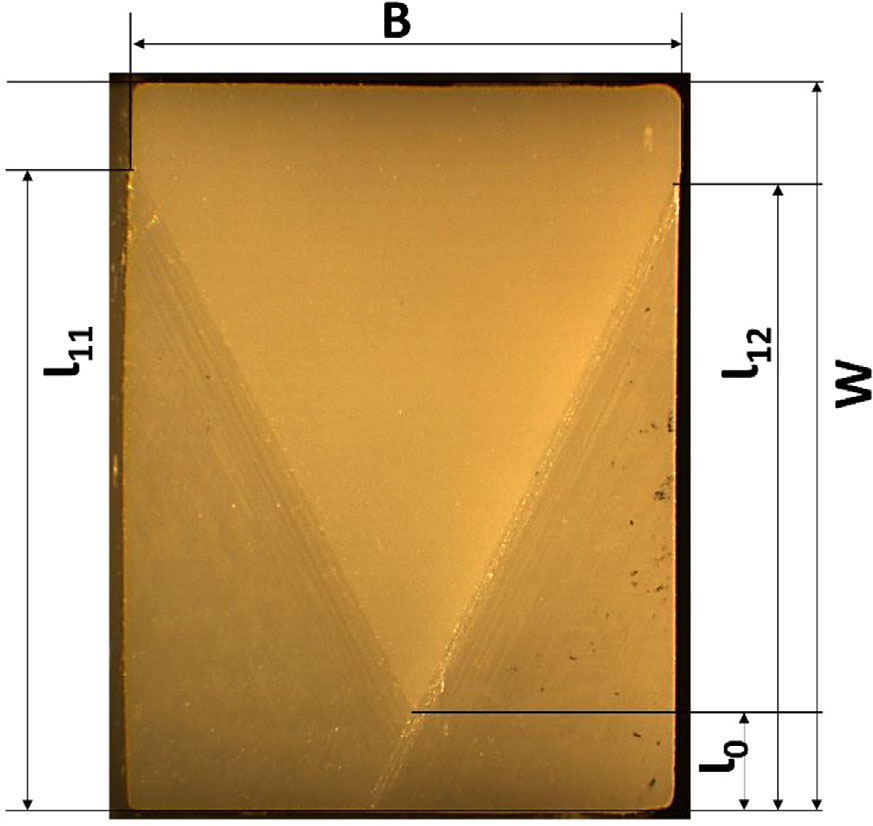
Chevron notch after fracture toughness test. B = specimen thickness; W = specimen width; ɭ_11_&ɭ_12_ = chevron dimensions; ɭ_0_ = chevron tip dimension.

After the test, the chevron notch was examined at 30 × magnification under an optical microscope for measurements (Figure 3). The fracture toughness (*K*_Ic_) was calculated using the following formula:

$$K_{IC}=\;{Y^{*}}_{min}\left[\frac{F_{max}\left(S_{0}-S_{i}\right)}{BW^{1.5}}\right]\frac{1}{\sqrt{1000}}$$

$${Y^{*}}_{min}=\frac{0.3874-3.0919\alpha_{0}+4.2017\alpha_{1}-2.3127\alpha_{1}^{2}+0.6379\alpha_{1}^{3}}{1.000-2.9686\alpha_{0}+3.5056\alpha_{0}^{2}-2.1374\alpha_{0}^{3}+0.013\alpha_{1}}$$

where *B* is the specimen thickness, *W* is the specimen width, *F*_max_ is the maximum force applied to the test specimen by the test machine, *S*_0_ is the flexure fixture outer span, *S_i_* is the flexure fixture inner span, Y^*^_min_ is the stress intensity factor coefficient minimum value. *α_0_* = *(Ɩ_0_|W)*, where *Ɩ_0_* is the chevron tip dimension, and *α_1_* = *(Ɩ_1_|W)* where *Ɩ_1_* is the average of *Ɩ_11_* and *Ɩ_12_* (chevron dimensions). The measurements are represented in Figures 2b and 3.

### Microhardness measurement

After completing the fracture toughness tests, ten 3Y‐TZP specimens from each of the nonaged and aged groups were then polished with silicon carbide papers ranging from 600 to 4000‐grit (Metaserv 250 Grinder Polisher; Buehler) at a speed of 350 rpm under running water. The specimens underwent microhardness testing using the microhardness tester (FM‐700, Future Tech, Kawasaki, Japan). A force of 1 kg with a 20‐s dwell period was applied using a pyramid‐shaped indenter point. Five indentations were made on the same surface side of each specimen, maintaining a minimum separation of 1 mm between each indentation. Following this, the mean Vickers hardness number (VHN) was calculated for each group of specimens.

### Microstructure analysis and phase characterization

A specimen from each of the 3Y‐TZP 3D‐printed and milled groups (nonaged and aged) was randomly selected for surface topography analysis. This analysis was performed on the polished surface of the chosen specimens using SEM (Philips XL 30 CP, Philips, Eindhoven, Netherland). The SEM was equipped with EDX to determine the chemical composition of the specimens (nonaged and aged) represented by the weight percentage (wt.%) of each element. The specimens were coated with a thin layer of gold‐palladium and subsequently examined at a magnification of 1000 × using a secondary electron detector operating at an acceleration voltage of 12.0 kV.

XRD was utilized to assess the composition of the zirconia phase and determine any phase transformations. A specimen from each of the 3D‐printed and milled groups (nonaged and aged) was analyzed using an X‐ray Diffractor (model‐Discover D8, Bruker‐Germany) with Cu kα radiation (wavelength 1.542 A°). The peaks were measured within a range of 20°–90° at 5 s per step and a step size of 0.02°.

### Statistical Analysis

The data for the fracture toughness and hardness were checked to ensure normal distribution (*p* > 0.05), as indicated by the Shapiro–Wilk and Kolmogorov–Smirnov tests. The effects of the manufacturing technologies (3D‐printed vs. milled technologies) and the aging process (nonaged vs. aged specimens) on fracture toughness and hardness were analyzed using two‐way ANOVA and Student *t*‐tests (*α* = 0.05). All statistical analyses were performed using statistical software (IBM SPSS Statistics, v29.0; IBM Corp).

## RESULTS

The outcomes of the two‐way ANOVA indicated that the material type (*p* < 0.001), the aging procedure (*p* < 0.045), and their interactions (*p* = 0.033) significantly affected the fracture toughness values (Table 1). Conversely, the material type (*p* = 0.096), the aging procedure (*p* = 0.795), and their interactions (*p* = 0.469) did not significantly affect the hardness values (Table 1). The means and standard deviations (±SD) of fracture toughness and Vickers hardness values are presented in Table 2. There were no significant differences in hardness values between any tested groups (*p* > 0.05). As can be observed in Table 2, both aged and nonaged 3D‐printed groups exhibited higher fracture toughness values than milled groups. Moreover, the fracture toughness value (6.07 ± 1.10 MPa m^1/2^) of the specimens in the nonaged 3D‐printed group was significantly (*p* < 0.05) higher than those in the milled groups (nonaged 4.46 ± 0.16 MPa m^1/2^ and aged 4.50 ± 0.08 MPa m^1/2^). However, the process did not significantly impact the fracture toughness values of the specimens produced through any of the manufacturing techniques.

**TABLE 1:** Two‐way ANOVA results for fracture toughness and Vickers hardness measurements.

Test	Source of variation	Sum of squares	df	*F*	*p*
Fracture toughness	Material type	11.330	1	18.958	0.001^*^
	Aging procedure	2.567	1	4.295	0.045^*^
	Material type × Aging procedure	2.923	1	4.891	0.033^*^
Vickers hardness	Material type	2556.641	1	2.913	0.096
	Aging procedure	60.148	1	0.069	0.795
	Material type × Aging procedure	469.568	1	0.535	0.469

df, degree of freedom, F: ratio of two variance, *p*: probability of error.

*statistically significant (*p *< 0.05).

**TABLE 2:** Means and standard deviations (±SD) of fracture toughness and Vickers hardness values using *T*‐test.

Group	Subgroup	Fracture toughness (MPa m^1/2^)	Vickers hardness (N/mm^2^)
3D‐printed	Nonaged	6.07±1.10^a^	1422.76±16.49^a^
	Aged	5.02±1.07^ab^	1413.45±31.78^a^
Milled	Nonaged	4.46±0.16^b^	1431.90±32.07^a^
	Aged	4.50±0.08^b^	1436.10±34.62^a^

Different superscript letters (a,b) indicate significant differences (*p <* 0.05) between subgroups within materials.

Similar superscript letters (a,b) indicate no statistically significant differences (*p > *0.05) between subgroups within materials.

**FIGURE 4:**
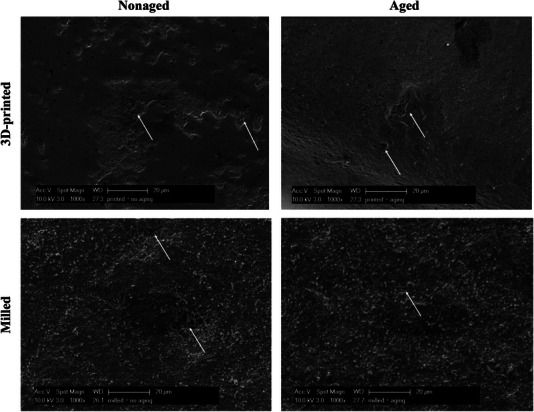
Representative SEM images for the tested groups with magnification of 1000×. Arrows point to the surface flaws.

SEM images revealed surface variations among the subgroups in Figure 4. The milled 3Y‐TZP specimens exhibited rougher surfaces than the 3D‐printed specimens. Surface flaws were observed in both milled and printed specimens. Aging did not appear to have a significant effect on the surface. The EDX analysis revealed the 3D‐printed and milled 3Y‐TZP specimens had similar chemical compositions, as shown in Table 3.

**TABLE 3:** Surface elemental composition of 3D‐printed and milled 3Y‐TZP specimens (nonaged and aged) provided by EDX.

Group	Subgroup	Elements				
		Zr wt.%	O wt.%	Y wt.%	Hf wt.%	Al wt.%
3D‐printed	Nonaged	66.1	27.4	3.5	2.9	0.2
	Aged	66.2	28.2	3.4	1.8	0.4
Milled	Nonaged	66.5	26.6	4.2	2.4	0.3
	Aged	64.8	29.1	3.1	2.7	0.3

Zr = zirconium; O = oxygen; Y = Yttrium; Hf = Hafnium; Al = aluminum.

**FIGURE 5:**
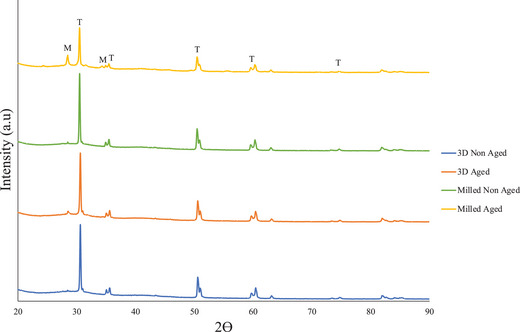
XRD diffraction patterns of the tetragonal (T) and monoclinic (M) phase of nonaged and aged groups.

XRD analysis revealed that both 3D‐printed and milled 3Y‐TZP exhibited similar phase patterns, predominantly consisting of tetragonal phases with minor monoclinic phases (Figure 5). During the aging process, an increase in monoclinic peaks and a slight decrease in tetragonal peaks were observed; however, the phase transformation was not particularly pronounced.

## DISCUSSION

The results indicate that autoclave aging has no significant impact on the hardness of both 3D‐printed and milled 3Y‐TZP (*p* = 0.795). The fracture toughness of nonaged 3D‐printed 3Y‐TZP was significantly greater than that of both aged and nonaged milled 3Y‐TZP. However, the fracture toughness of the aged 3D‐printed 3Y‐TZP was equivalent to that of aged and nonaged milled 3Y‐TZP. Consequently, the findings are in partial agreement with the null hypothesis. While these results are statistically significant with respect to fracture toughness, their clinical significance should be carefully considered. Statistical significance means the observed differences are unlikely due to random chance, but this does not automatically imply clinical relevance. Clinical significance involves the practical impact on patient outcomes and treatment effectiveness. Differences in fracture toughness may indicate variations in material performance, but their real‐world impact on the longevity of dental restorations and patient outcomes requires further investigation through extensive clinical trials. Thus, while the statistical findings are promising, their clinical importance remains to be fully validated.

Milling is the most frequently applied method for the production of zirconia‐based restorations.^1^ However, additive manufacturing is making consistent progress within the field of prosthetic dentistry.^21^ One of the recognized technologies used in AM is vat polymerization, which includes stereolithography (SLA) and direct or digital light processing (DLP).^13^ Dental crowns and bridges manufactured with SLA and DLP have finer surface polish and greater precision than those manufactured using material jetting (MJ) and robocasting (RC), which generate crowns and bridges with inferior mechanical strength.^14^ There is an increasing focus on research to improve the mechanical performance and dimensional accuracy of zirconia produced using 3D printing. Despite the introduction of the most recent LCM (Lithoz, Vienna, Austria) technology, the impact of aging on this innovative 3D‐printed 3Y‐TZP has not been sufficiently studied.

Autoclaving is a frequently applied aging method to facilitate our understanding of the long‐term behavior of zirconia‐based materials in vivo.^22^ Aging in an autoclave at 134°C for 1 h theoretically corresponds to 3–4 years of clinical service.^23^ The 5 h aging period applied in this investigation may have provided a more comprehensive view of the aging characteristics of this unique material. The effect of accelerated aging could be seen as material degradation, which can be examined directly by surface analysis using scanning electron microscopy and profilometer or indirectly through measuring the changes in hardness and mechanical strength.^24^ Moreover, zirconia could transform between monoclinic, tetragonal, and cubic phases. These transformations could be analyzed using X‐ray diffraction.^2^ In this study, the fracture toughness, hardness, SEM, EDX, and XRD were all employed to ascertain the impact of aging on zirconia generated via LCM versus the milled type.

The fracture toughness of a material is a quantitative measure that indicates how resistant it is to the propagation of an artificially generated crack before catastrophic failure.^25^ It was established that fracture toughness correlates significantly with in vivo fracture resistance and is a significant predictor of clinical durability.^26^ The fracture toughness could be assessed using well‐established tests such as single‐edge‐V‐notch beam (SEVNB), compact tension (CT), double torsion, and chevron notch beam (CNB) tests.^27^ Notch‐less triangular prisms (NTP) and modifications have emerged as recent advancements in testing methodology.^28^ A recent inter‐laboratory round robin research supports the application of CNB as a viable alternative for SEVNB in the precise determination of fracture toughness of fine‐grained zirconia milled blocks.^29^ This endorsement is contingent upon adherence to the ISO 24370:2005 standard.^19^ The CNB technique yielded a reported range of 4.55 to 6.22 MPa m^1/2^ for zirconia in one investigation.^30^ Another study employed CNB showed comparable fracture toughness values for 3D‐printed and milled 3Y‐TZP (4.17 ‐ 4.71 MPa m^1/2^).^31^ Overall, the fracture toughness values for DLP were shown to be in the range of 3.43 to 6.42 MPa m^1/2^ similar to those of conventionally fabricated zirconia.^6^, ^32^ These findings are consistent with the *K*_Ic_ results obtained in the current study of 3D‐printed and milled 3Y‐TZP, which ranged from 4.46 to 6.07 MPa m^1/2^. Moreover, the round robin study^29^ identified two primary determinants that significantly impacted the *K*_Ic_ results: the storage media used and the chevron notch processing, which comprised notch geometry and cutting offset. In the current study, autoclave aging did not significantly affect the fracture toughness of the specimens using the CNB technique in both the 3D‐printed and milled 3Y‐TZP.

The investigated specimens exhibited a hardness range of 1413–1436 VHN, with no statistically significant distinction observed between the two materials nor the aged and nonaged specimens. Consistent with the aforementioned findings, the hardness of DLP‐sintered components varied between 1189 and 1556 VHN.^6^ These values were similar to those observed in conventionally manufactured zirconia and monolithic blocks.^33^ Numerous contributing aspects have been investigated through experimental research, including the association between an increase in ZrO_2_ load content and enhanced mechanical resistance characterized by reduced surface cracks and pores.^34^, ^35^ One investigation revealed that an increase in the proportion of zirconia could lead to a decrease in hardness.^33^ The present study findings for the surface elemental analysis showed similar chemical compositions of Zr in 3D‐printed and milled 3Y‐TZP, which ranged from 64.8 to 66.5 wt.%.

There is still inconclusive evidence about the effect of aging on the mechanical properties of materials composed of zirconia. The mechanical strength of the specimens fabricated through subtractive manufacturing was significantly reduced after aging in an autoclave at 134°C under 0.2 MPa pressure for 5 h.^36^ Other research found that thermal cycling reduced the hardness but not the fracture toughness for milled zirconia blocks.^31^, ^37^ In contrast, aged pre‐sintered specimens milled from monolithic zirconia blocks exhibited no discernible impact on their roughness, microhardness, or flexural strength. However, their phase transition was markedly altered.^38^ The mechanical properties of other specimens did not significantly differ when aging temperatures or storage time were increased.^22^, ^39^

The method by which 3D‐printed 3Y‐TZP is produced has a substantial impact on its aging behavior. A study found that the mechanical resistance of DLP zirconia specimens was not significantly reduced after autoclaving for up to 15 h (134°C, 0.2 MPa), unlike the SLA specimens.^40^ However, both DLP and SLA showed increased monoclinic phase content correlating with aging time.^6^, ^40^ Similarly, the phase transformation for the 3D‐printed and milled 3Y‐TZP showed resistance to aging with no significant changes in the phase patterns other than an increase in the monoclinic peaks and a slight reduction in the tetragonal peaks. Reducing the transformation capacity of the zirconia was associated with lower mechanical resistance.^41^, ^42^ Increasing the monoclinic phase content correlated with rougher surfaces of 3Y‐TZP.^42^ Yet this increase in roughness was inconsistent among the studies.^6^, ^32^ SEM images (Figure 4) showed a rougher surface of milled 3Y‐TZP than the 3D‐printed alternative, however all specimens appeared smooth after aging, which might confirm the neglected effect of aging. Nevertheless, the mechanical characteristics of tested new 3D‐printed 3Y‐TZP generated via LCM remained remarkably constant, presenting stable mechanical properties following prolonged aging.

Limited research has been conducted to ascertain changes in fracture toughness and flexural strength sensitive to variations in the volume percent of zirconia.^21^ Furthermore, the strength properties of the 3D‐printed specimens were shown to be significantly impacted by the printing orientation (0°, 45°, and 90°).^43^, ^44^ These effects were further influenced by the experimental testing process and the load‐to‐printed surface orientation applied.^21^, ^45^ Therefore, examining the relationships between the printing orientation of these novel LCM zirconia and the load‐to‐fracture tests might have clinical implications.

## CONCLUSION

The artificial aging did not significantly affect the hardness of either the 3D‐printed or milled 3Y‐TZP. However, the fracture toughness of nonaged 3D‐printed 3Y‐TZP was found to be significantly higher than that of both aged and nonaged milled 3Y‐TZP, while the fracture toughness of aged 3D‐printed 3Y‐TZP was statistically equivalent to that of both aged and nonaged milled 3Y‐TZP. These findings suggest that LCM technology can produce 3D‐printed 3Y‐TZP with mechanical properties that are stable and comparable to those of milled zirconia, even after aging. Further research is needed to determine the clinical significance of these findings.

## CONFLICT OF INTEREST STATEMENT

The authors declare no conflict of interest.
